# Achalasia Secondary to Submucosal Invasion by Poorly Differentiated Adenocarcinoma of the Cardia, Siewert II: Consideration on Preoperative Workup

**DOI:** 10.1155/2014/654917

**Published:** 2014-07-15

**Authors:** Antonino Agrusa, Giorgio Romano, Giuseppe Frazzetta, Giovanni De Vita, Daniela Chianetta, Giuseppe Di Buono, Silvia Di Giovanni, Vincenzo Sorce, Gaspare Gulotta

**Affiliations:** Dipartimento di Chirurgia Generale d'Urgenza e dei Trapianti d'Organo, U.O.C. Chirurgia Generale e d'Urgenza, Policlinico Universitario “Paolo Giaccone,” Via Liborio Giuffrè 5, 90100 Palermo, Italy

## Abstract

Secondary achalasia due to submucosal invasion of cardia by gastric cancer is a rare condition. We report a case of pseudoachalasia, secondary to the involvement of gastroesophageal junction by poorly differentiated gastric cancer, initially mistaken as idiopathic form. We focus on the difficulty to establish differential diagnosis only on the basis of routine exams and we stress the necessity of “second level” instrumental exams; EUS in routine workup in selected patients should be considered. We support that routine workup based on history, clinical presentation, radiological and endoscopic findings, and certainly manometry could be insufficient for a correct differential diagnosis between primary and secondary forms in some patients.

## 1. Introduction 

Achalasia is a rare primary motility disorder of the esophagus that affects one person in 100.000 per year [1–3], consisting in absence of peristalsis and incomplete relaxation of frequently hypertensive lower esophageal sphincter (LES) in response to swallowing [4]. The disease was first described more than 300 years ago as “food blockage in esophagus” but the exact pathogenesis still remains enigmatic [5]. Achalasia can be primary [idiopathic] or secondary. The most common form of achalasia is the idiopathic one. Similar clinical presentation can occur in patients with pseudoachalasia (2–4% of patients with achalasia) or Chagas disease. Diagnosis is based on a careful clinical history, correct interpretation of anamnesis, and presentation of symptoms, barium swallow, endoscopy, and esophageal manometry. However, sometimes differential diagnosis might not be easy by “routine” methods in the initial evaluation as demonstrated by several cases [6–8], because clinical presentation can mimic idiopathic one; this often results in a substantial delay in diagnosis and treatment until surgery.

## 2. Case Report 

**Figure 1:**
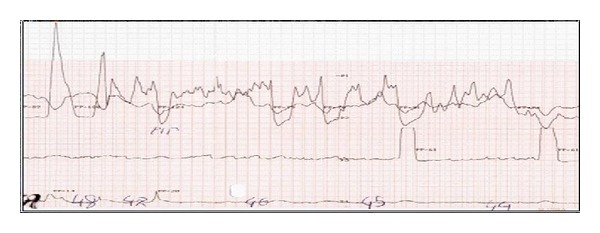
Manometry.

**Figure 2:**
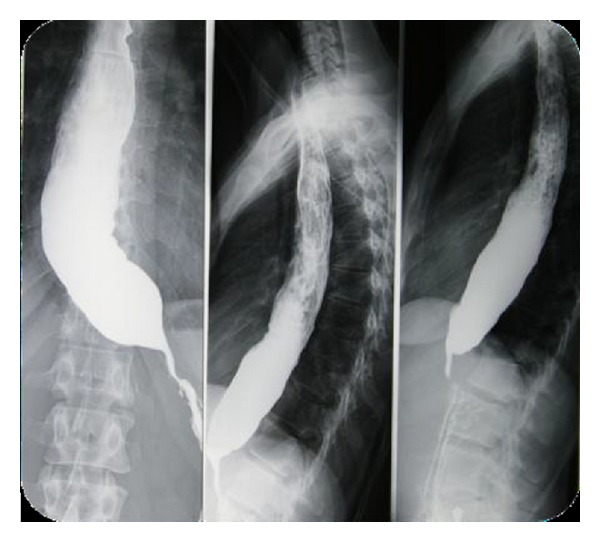
Barium swallow showing emptying defect with “bird beak” sign and narrowing at esophageal junction.

**Figure 3:**
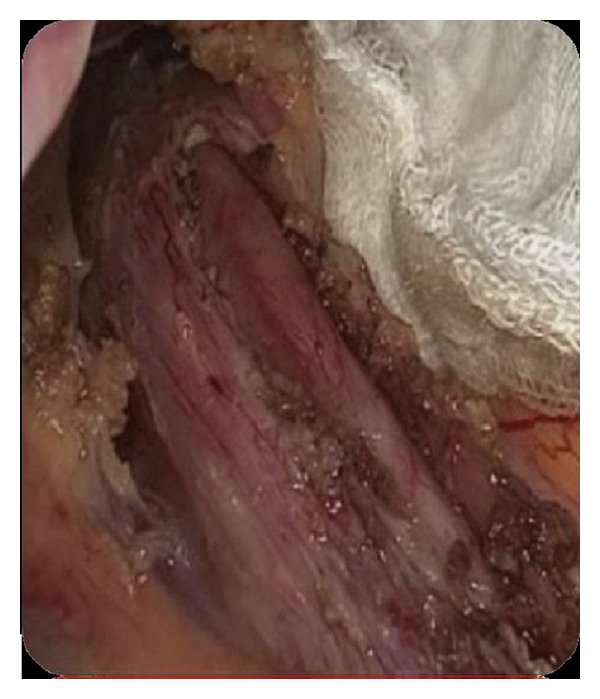
Laparoscopic Heller's myotomy.

**Figure 4:**
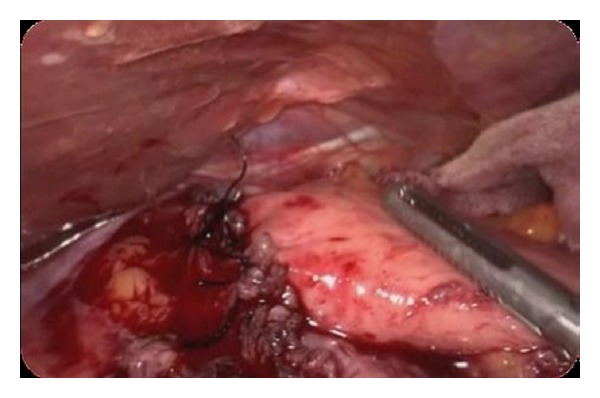
Dor fundoplication.

**Figure 5:**
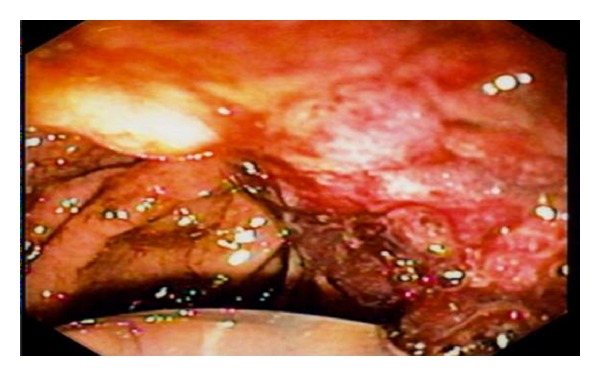
EUS.

**Figure 6:**
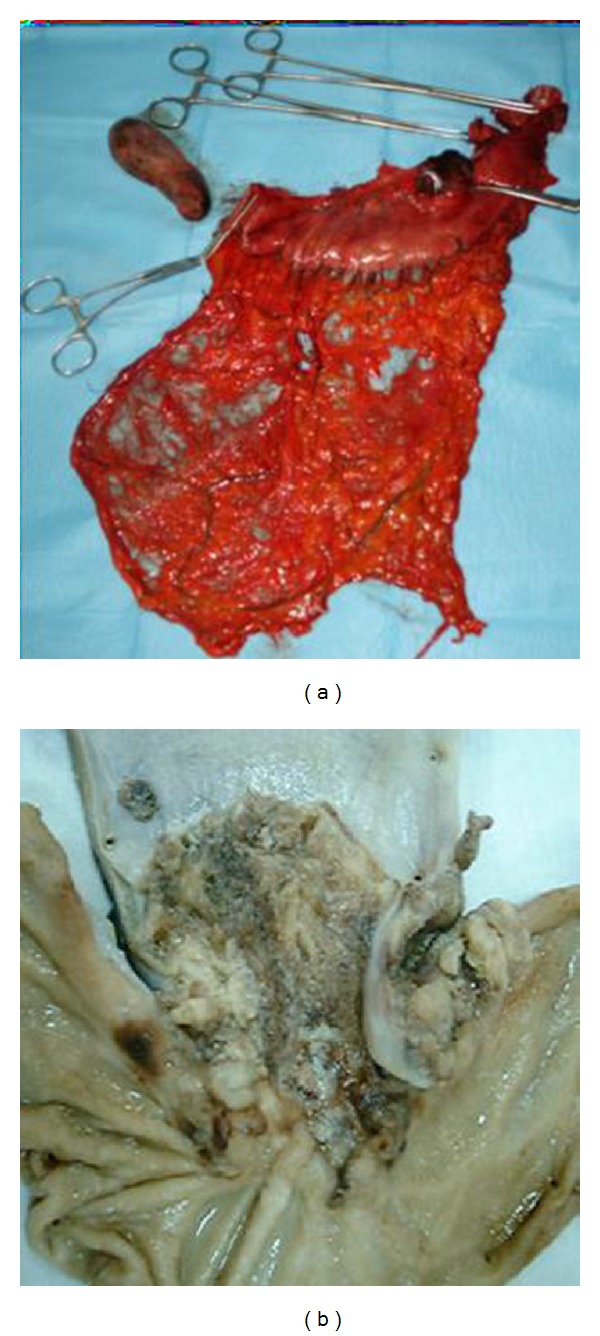
Specimens.

**Figure 7:**
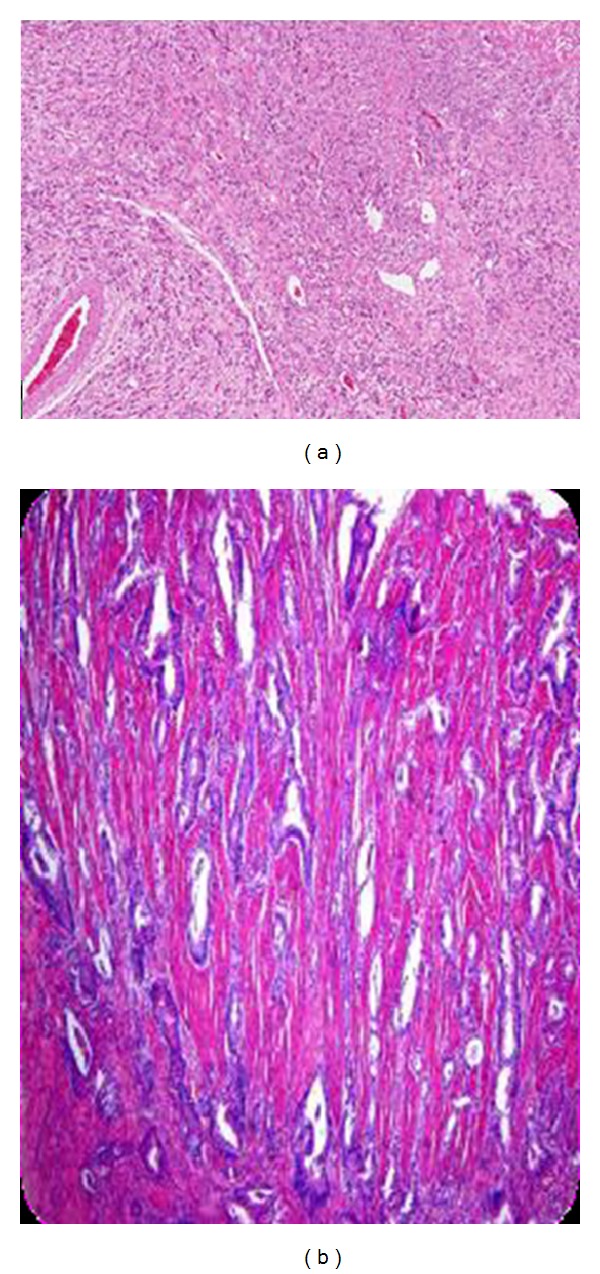
Histological finding was “poorly differentiated adenocarcinoma.”

A 51-year-old man suffered from progressive dysphagia to solids for over about 6 months and slow weight loss of 5 kg was referred to our institution with the presumptive diagnosis of achalasia. Preoperative workup consisted in a careful clinical history, correct interpretation of anamnesis, and symptoms; then esophagogastroduodenoscopy was performed and showed a dilated esophagus without evident mucosal lesion, and the passage through the cardia was more difficult: “crossing snap” passage. Manometry revealed absence of peristalsis and impaired swallow-induced LES relaxation with classic manometric pattern (Figure 1). The barium swallow showed an emptying defect with “bird beak” sign and narrowing at esophageal junction (Figure 2). We made a diagnosis of esophageal achalasia and performed a laparoscopic Heller's myotomy with Dor fundoplication (Figures 3 and 4). We use the laparoscopic approach to perform this kind of surgery. Several studies demonstrate that this is the best way to treat this pathology. We use Veres' needle placed in Palmer's point to create pneumoperitoneum and then we place five trocars: the first 10–12 mm trocar is placed in supraumbilical region, two 10–12 mm trocars in right and left side, and two 5 mm trocars in paraxiphoid region and left iliac region [9]. The surgical exploration of peritoneal cavity does not reveal any anatomic abnormality or suspect neoplastic lesions or secondary localizations. Two months after surgery, the patient complained of worsening dysphagia. We repeated several esophagogastroduodenoscopies with numerous biopsies that showed only cardia inflammation and were negative for malignancy. Tumor markers were negative too. Balloon dilatation was performed to improve symptoms. Persisting dysphagia, one month after we performed EUS with biopsies, showed infiltrating gastric adenocarcinoma of the cardia (T3 WHO, Siewert II) (Figure 5). After staging CT scan, total gastroesophagectomy was performed, with D2 lymph-node resection and Roux-en-Y reconstruction. The surgical specimen contained a thick, hard tumor 3,6 × 2,5 × 1,3 cms located in the gastroesophageal junction (Siewert II), extensively infiltrating the wall and reaching in some areas the subserosal layer (Figures 6(a) and 6(b)). Histological finding was “poorly differentiated adenocarcinoma (WHO: G3)” (Figures 7(a) and 7(b)).

## 3. Discussion 

The first description of this condition was made by Willis T. (1674), who treated these patients with dilatators made of whale bone and sponge. The term achalasia is a Greek word meaning “failure of relaxation” which was first coined by Hurst in 1927. Idiopathic form is caused by inflammation of myenteric plexus, injury and subsequential loss of inhibitory ganglion cells, and fibrosis of myenteric nerves. There is a reduction in the synthesis of nitric oxide and vasoactive intestinal polypeptide [10]. Several studies tried to identify the agents that may cause the disease; the etiology of primary achalasia is maybe an autoimmune-mediated destruction of inhibitory neurons in response to an unknown insult, in genetically susceptible ones. However, the exact pathogenesis of primary achalasia is still poorly known; a definite “trigger” has not been identified [11]. Pseudoachalasia is a clinical syndrome due to several malignant tumors, which can mimic idiopathic form. The pathophysiology of pseudoachalasia is still not well understood too. Different theories have been proposed: intraluminal narrowing, extra luminal compression, infiltration of the myenteric plexus, metabolic disturbance, and paraneoplastic effects as endocrine abnormalities (e.g., hypercalcaemia) that may result in interference on the controls and coordination of neural plexus on the swallowing mechanism or in vagal neuropathy and subsequent degeneration of plexus ganglia [12, 13]. The most common cause of pseudoachalasia is the compression of esophagus by an extraluminal [14, 15] mass; 71% of the cases result from a gastric adenocarcinoma at the gastroesophageal junction [16, 17]. Usually, in these cases the esophagogastric junctional mucosa remains intact, without superficial macroscopic lesion; the mass may have the appearance of a benign intramural lesion [17]. However several malignancies could be cause of this disorder such as gastric adenocarcinomas, bronchial origin adenocarcinoma and oat cell tumors, squamous cell carcinoma of the esophagus, Hodgkin/Non-Hodgkin's lymphoma, pleural mesothelioma, hepatocellular carcinoma, prostatic adenocarcinoma, breast adenocarcinoma, colonic adenocarcinoma, pancreatic adenocarcinoma renal cell carcinoma, and squamous cell carcinoma of the cervix [18]. The myenteric plexus may be infiltrated directly or attacked by host's immune system that creates autoantibodies in response to surface tumour antigens that can cross-react leading to nerve injury and damage [19, 20] Achalasia has also been reported due to antineuronal antibodies (anti-Hu, anti-Yo, and N-type Ca^2+^ channel antineuronal antibodies) especially in patients with small cell lung cancer [21, 22]. Achalasia can also be part of complex syndrome such as Allgrove syndrome, Down's syndrome, or familial visceral neuropathy [23]. Differential diagnosis is often difficult, but it is mandatory because pseudoachalasia is due to neoplasms of the gastroesophageal junction: it should be excluded.

Progressive dysphagia to solid followed by liquids is the most characteristic symptom of primary achalasia [24, 25]. However, dysphagia in achalasia secondary to malignancy is relatively short in duration and usually develops in advanced phases of pathology. Although a secondary form of achalasia due to esophageal or gastric cancer involving the cardia could be easily diagnosed through upper gastrointestinal endoscopy, if the malignancy spreads into submucosal layer or is a very aggressive and poorly differentiated histological type, it can be very difficult to diagnose it until surgery [26].

In a literature review a total of 264 cases of pseudoachalasia were found in 122 publications [27]. Most cases of these were due to malignant disease (53.9% primary and 14.9% secondary malignancy), followed by benign lesions (12.6%) and complications of surgical procedures at the distal esophagus or proximal stomach (11.9%). Rarely, the disease was consequence of paraneoplastic process with destruction of the myenteric plexus (2.6%).

In a more recent research, other authors report 155 publications with data of 302 patients diagnosed with pseudoachalasia [28]. These studies show that primary malignancies of the esophagus or esophagogastric junction accounted for 50% of cases of secondary achalasia, followed by secondary malignancies (18%), such as metastases (12%), which primarily originated from lung and breast. Benign causes, including mesenchymal tumors, secondary amyloidosis, and peripheral neuropathy, accounted for 14% of patients with pseudoachalasia. In 12%, the motor abnormality occurred as a consequence of gastroesophageal surgery, namely, antireflux surgery. Rare causes of pseudoachalasia were neurological disorders (3.5%) or paraneoplastic syndromes (2.5%) in the context of small cell carcinoma, bronchial carcinoid, gastric carcinoma, and pleural mesothelioma. However, none of these paraneoplastic syndromes was associated with mediastinal or esophageal infiltration by the primary tumor.

## 4. Conclusion

Secondary achalasia due to malignant disease is an infrequent disorder. It should be suspected in elderly patients who have had a significant weight loss but a short duration of symptoms. Pseudoachalasia represents a significant diagnostic challenge, with clinical, radiological, manometric, and endoscopic features that may be indistinguishable from achalasia. As reported in this unfortunate case, multiple diagnostic procedures may lead to inappropriate diagnostic conclusion of a benign etiology. When the clinical presentation suggests a malignancy that cannot be identified by routine evaluation, an endoscopic ultrasound and then a fine CT scan should be performed. EUS could be the “keystone” to diagnostic success, with attributes of high image resolution with close proximity to the lesion, compared with CT and magnetic resonance imaging which may lack sensitivity for small lesions. EUS also facilitates acquisition of a tissue sample, adding further to its utility in the diagnostic workup of patients in which pseudoachalasia is suspected. EUS represents a valuable tool in the evaluation of pseudoachalasia, particularly where clinical suspicion is high and other modalities have been unrevealing [29, 30]. Considering the patient's age, the lack of evidence for malignancy in the clinical history, and the findings in the first endoscopy and biopsies, we argue that it may be appropriate to amend the preoperative workup for achalasia introduction of EUS among routine exams to reduce the delay in diagnosis.
